# Real-World Effectiveness of the Varicella Vaccine among Children and Adolescents in Qatar: A Case–Control Study

**DOI:** 10.3390/vaccines11101567

**Published:** 2023-10-05

**Authors:** Zahra Bibi, Ahmed Daniyal Nawaz, Maha Al Kurbi, Shahad Fakhroo, Khaled Ferih, Noor Al-Jaber, Merin Alex, Khalid H. Elawad, Tawanda Chivese, Susu M. Zughaier

**Affiliations:** 1 College of Medicine, QU Health, Qatar University, Doha P.O. Box 2713, Qatar; zb1806504@student.qu.edu.qa (Z.B.);; 2 Health Protection, Primary Health Care Corporation (PHCC), Doha P.O. Box 26555, Qatar

**Keywords:** varicella-zoster virus, chickenpox, varicella vaccination, vaccine effectiveness, Qatar

## Abstract

Background: Despite the availability of a highly efficacious vaccine, varicella outbreaks are still being reported globally. In this study, we evaluated the real-world effectiveness of varicella vaccination among children between the ages of 1 and 18 years old during the period 2017 to 2019 in Qatar. Methods: A matched case–control study was conducted that included all reported varicella-infected children who visited the primary healthcare system in Qatar from January 2017 to December 2019. The cases were children under the age of 18 years who were clinically diagnosed with varicella. The controls were of the same age, who visited the Primary Health Care Corporation (PHCC) during 2017–2019 with a skin rash where varicella infection was ruled out. The data on varicella vaccination for each participant were obtained from the electronic database in the PHCC during the study period. Results: We included 862 cases of varicella and 5454 matched controls, with a median age of 8 years (IQR 3–12); 47.4% were female and almost 50% were of Qatari nationality. The year 2019 had the highest varicella infection count with a total of 416 cases. The cases were less likely to be vaccinated against varicella, with approximately a quarter (25.6%) of cases and 36.7% of the controls having either one or two doses of the vaccine (*p* < 0.001). Compared to not being vaccinated, a single dose vaccination showed a 56% reduction in the odds of varicella infection [OR 0.44, 95% CI: 0.34–0.55; *p* < 0.000], and a two-dose vaccination showed an 86% reduction in the odds of varicella infection [OR 0.13, 95% CI: 0.06–0.29; *p* < 0.000]. Conclusion: In this multicultural setting, a two-dose varicella vaccination shows reasonable protection against varicella infection.

## 1. Introduction 

Varicella infection is an acute and highly contagious disease caused by varicella-zoster virus (VZV), which belongs to the Herpesviridae family and naturally only infects humans [1]. The currently licensed varicella vaccine is a live attenuated viral formulation available in two forms: a single antigen and in combination with measles, mumps, and rubella [2]. After the introduction of the VZV vaccine by the WHO, most developed countries and some developing countries successfully included it in their national vaccination programs. Currently, two live attenuated VZV-containing vaccines for the prevention of varicella are licensed for use in the United States [3]. The VAR (Varivax) vaccine is a live attenuated vaccine formulation and the MMRV (ProQuad) vaccine is a combination measles, mumps, rubella, and varicella vaccine [4]. In Middle Eastern countries, such as Qatar, Saudi Arabia, UAE, Kuwait, and Bahrain, VAR (Varivax) vaccine (marketed as Varilrix^®^) is the preferred vaccine. After its introduction in Qatar at PHCCs in 2002, it was finally considered as part of the mandatory national vaccination program in 2007 [5].

The safety of both the single-antigen varicella vaccine (VAR) and the combined measles, mumps, rubella, and varicella vaccine (MMRV) has been extensively studied. Analyzed data from the United States Vaccine Adverse Event Reporting System (VAERS) from 2006 to 2020 found that the vaccines exhibited favorable safety profiles when administered as recommended. The side-effects (SEs) of the VAR vaccine primarily involve injection site discomfort, localized reactions, rash, and fever, with approximately 4.1% of the reported SEs classified as serious. Similarly, the most common adverse health events after both VAR and MMRV were injection site reactions (31% and 27%), rash (28% and 20%), and fever (12% and 14%), respectively [6]. Moderate SEs involve fever-induced seizures and respiratory symptoms, while severe reactions like pneumonia and brain issues are extremely rare and their connection to the vaccine is uncertain [7,8]. While the identified adverse events are generally in line with expected patterns, no new or unexpected safety concerns emerged for either vaccine, reinforcing their overall safety credentials. These results underscore the importance of continuous safety surveillance and transparent communication in maintaining public confidence in vaccination [9].

In newborns, some varicella infection may be due to congenital infection, although this is very rare with an incidence of 0.59% [10]. Congenital varicella syndrome (CVS) is a rare but serious multi-system disorder due to infection by the VZV. It occurs when the mother is infected during the first half of pregnancy, with week 13 to 20 of gestation being the critical period with the greatest likelihood of transmission.

In Qatar, varicella infection is the second most common notifiable disease, although the vaccine coverage rate is currently estimated at 95%, one of the highest rates in the world [11,12]. According to Qatar’s pediatric vaccination schedule (Supplementary Figure S1), two doses of varicella vaccine are provided for free, the first dose is given at 1 year of age and the second dose is given between 4 and 6 years of age [13].

Despite the availability of highly efficacious vaccines, the global incidence of varicella from 1990 to 2020 saw an upward trajectory. The highest burden was specifically in the younger age group (5 and below) and the elderly population. The highest increase was noted in the high-income areas in the Asia-Pacific region. Some of the reasons for this could be due to better detection tools and infectious disease surveillance systems in the developing world. Furthermore, the increasing elderly population worldwide may also play a role. The disability-adjusted life year (DALY) and death due to varicella has decreased over the past 3 decades. This could be due to early detection, advancements in the management of varicella, and the global vaccination roll out for varicella [14].

Varicella vaccine effectiveness is defined as the measure of protection the vaccine administration provides under a certain field of conditions to a particular population [15]. Several studies have been conducted to calculate vaccine efficacy, including seroprevalence studies, modeling studies, observational studies, databases, and case–control studies. However, the most numerous and beneficial have been from outbreaks studies as this has helped scientists in predicting the real-world effectiveness of the vaccine in which the ideal situation for the vaccine administration program is never perfect [16].

The varicella vaccine effectiveness for one dose of varicella vaccine against any disease ranges from 55% to 87%, while after two doses, the vaccine effectiveness ranges between 84% and 98% [17,18]. As for the effectiveness of VAR (Varivax), which is the vaccine that has been implemented in Qatar and many other Middle Eastern countries, a meta-analysis of post-licensing studies with different designs including mainly retrospective and prospective cohort studies, and matched case–control studies found the effectiveness of one dose of varicella vaccine to be 82% against any clinical varicella and 98% against moderate to severe disease. Two doses of vaccine demonstrated 92% effectiveness against any clinical varicella [19].

This vaccination program has tremendously reduced the incidence rate and the burden of disease. In the U.S., the program was implemented in 1995 and the number of cases went from more than 4 million cases per year to less than 150,000 cases per year as per the recent data in 2022. This resulted in a significant decrease in the number of hospitalizations and deaths per year from varicella infection [20]. However, surveillance data regarding varicella epidemiology, vaccination status, seroprevalence, and the real-world effectiveness of the vaccine is still lacking in the Middle East [5]. Thus, more data must be provided regarding the rates of infection, along with the vaccination status of these infected persons. This way, we can predict the national coverage of the vaccine and the efficacy of the vaccination program in each country, its impact on disease incidence, morbidity, and mortality, along with evaluating the real-world effectiveness of the varicella vaccine itself under the imperfect conditions of routine use. Finally, this research can provide more information regarding the cause of the vaccine and/or the vaccination program failure, and possible solutions to tackle the underlying issues and obstacles.

This study aims to evaluate the real-world effectiveness of varicella vaccination among children and adolescents in Qatar.

## 2. Materials and Methods

### 2.1. Study Design

This study was a matched case–control study design where cases and controls were selected from the PHCC electronic database in Qatar based on varicella disease status. This design has been used previously in studying real-world vaccine efficacy in the absence of viable prospective and retrospective cohorts [21,22,23]. Further, the matched case–control study is an efficient design which reduces the time and resource costs that are required in a cohort. The matching in this design enables the study to address confounding factors at the design stage, thereby limiting false inferences especially in the descriptive sections of a study’s report.

### 2.2. Matching of Cases and Controls

We matched cases to control at a ratio of 1:7, respectively, using exact matching for the variables age, gender, and nationality. This means that cases were matched to controls who were of the exact age in years, biological sex, and nationality. Each case was matched to 7 controls or until all eligible controls were exhausted if there were less than seven. However, each case was matched to at least four controls.

### 2.3. Study Setting

The study included participants who visited the primary health care system from the 1st of January 2017 to the 31st of December of 2019. The health system in Qatar consists of public and private health systems. The public health system cares for the majority of the population (72%) and consists of a large comprehensive primary health care system run by the Primary Health Care Corporation (PHCC). The Primary Health Care Corporation (PHCC) under the umbrella of the Ministry of Public Health are the major free-of-charge health care providers in Qatar. Currently, there are 28 centers operating under the PHCC, providing 89 different services such as mental health, women’s health, oral hygiene, etc.

Qatar has a heterogeneous population that varies in ethnicity, education, knowledge, and experiences. According to the planning and statistics authority, in April 2023, the population of Qatar was 2,956,000 [24]. Twelve percent of them are Qataris, and the rest are from different nationalities such as Indian, Nepalese, etc. Furthermore, the demographic distribution is such that each area has a variety of nationalities all living in close proximity to each other and accessing their local health center. As of 2021, there were 2.1 million patients registered with the PHCC, which accounts for 72% of Qatar’s population. The primary health care centers are located in different localities all around Qatar and cater to the patients in their surrounding geography. Although the PHCC is largely state funded, individuals are required to pay a nominal QAR 100 (equivalent to USD 27.00). From the year 2012, all medical records are captured using an electronic health system set up by the Cerner Corporation.

Cerner is an American organization that supplies health IT services and devices to health corporations all around the world [25]. It helped Hamad and PHCC in 2012 to set up a nationwide electronic health system (EHS), also known as a clinical information system. The EHS allows a health corporation to digitally chart the patient’s medical records, prescriptions, etc. Through the Cerner system, any patient’s medical records can be accessed by any authorized health staff in any of the hospitals under Hamad Medical Corporation (HMC) and any of the health centers. Since 2012, Hamad and PHCC have completely digitalized all of their medical records [26].

In regard to Qatar’s strategy for varicella outbreaks, the current policy is to conduct contact tracing for all the confirmed varicella cases within 48 h, in alignment with the CDC’s recommendation. For example, in a school, all the classmates of an infected student will be considered as close contacts and must be referred to a primary healthcare center. Family members within the same household are also considered close contacts. Close contacts undergo prophylaxis based on their immunization history.

Study population and sampling strategy: Eligible participants in this study were children and adolescents aged between 1 and 18 year(s). Cases were children and adolescents who were diagnosed with varicella infection at any time during the period of 2017–2019. The diagnosis of varicella infection was made based on the clinical characteristics of the lesions, as per clinical guidelines [27]. No serology was used in the diagnosis of the participants. The controls were defined as children and adolescents between the age of 1–18 who visited the PHCC between 2017 and 2019 with a skin rash and had varicella infection ruled out.

### 2.4. Data Collection

Demographic data: The demographic information included gender, date of birth, nationality, and PHCC name.

Varicella vaccination data: For the children and adolescents vaccinated in Qatar before 2018, all the vaccination records were registered and collected on paper by the family physician. Since 2016, the PHCC has adopted an electronic system where all vaccinations are directly entered online. All the data are then stored on the Cerner system, the patient database used by the PHCC. For the expatriate children who were not born in Qatar, if they possessed an immunization card, the information on it was used to complete their vaccination form. If they did not possess an immunization card, their vaccination record was left empty.

### 2.5. Statistical Analysis

The categorical data are presented as frequencies and percentages. We assessed the distribution of age using histograms and then reported the median (IQR) for age as it was not normally distributed. For the variable age, which is a skewed continuous variable, we used the Wilcoxon rank-sum test to compare between the matched cases and controls. Categorical variables, including age groups, gender, vaccination status, visit year, and the nationality, were compared using the Pearson’s chi-squared test.

To investigate the association between vaccination status and risk of varicella infection, we used conditional logistic regression with varicella infection (yes/no) being the outcome. We reported odds ratios (ORs) and their 95% confidence intervals (95% CI), and exact *p*-values. Exact P-values were interpreted as evidence against the null hypothesis. To adjust for confounding, we adjusted for age and gender. All analyses were performed using Stata version 16 (College Station, TX, USA).

## 3. Results

### 3.1. Characteristics of Participants

During the study period, the PHCC reported 862 clinical cases of varicella infection who were then matched to 5454 individuals in the control group (Table 1). The cases were divided into three age groups: group 1 (1–5 years), group 2 (6–10 years), and group 3 (11–18 years); the number of cases in each group was 322 (37%), 249 (29%), and 291 (34%) cases, respectively. The majority of cases were in group 1 and most of them were in the first year of life with males (52.6%) being slightly more than females (47.4%). Because of the matched design, there were no differences between cases and controls in age, gender, and nationality. However, the percentage of all non-Qatari cases (53%) was higher than that of Qataris (47%).

### 3.2. Varicella Outbreaks during the Study Period

During the study period, the year 2019 had the highest varicella infection rate with a total of 416 cases out of a total of 862 cases in 2017–2019 (Supplementary Table S1). This was followed by the years 2018 and 2017 with 383 and 63 cases, respectively.

### 3.3. Age Distribution of Cases

**Figure 1:**
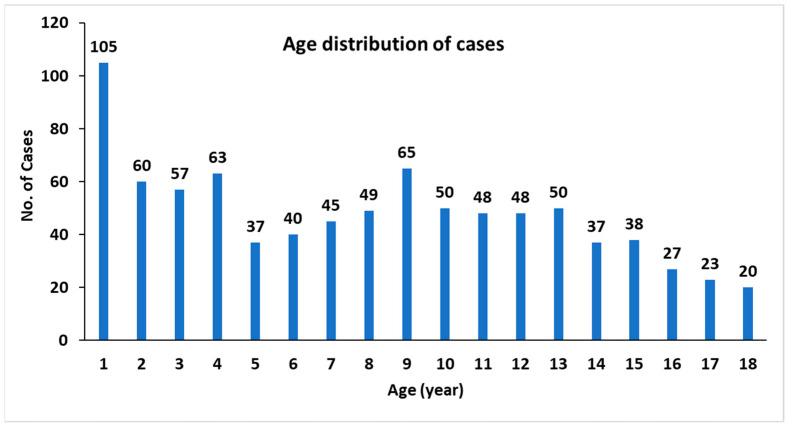
Number of cases reported by age among all PHCC centers between 2017 and 2019.

In the distribution of cases among ages, the greatest number of cases was seen in the children in their first year of life. The age group 1–5 (group 1) had the highest number of cases of varicella infection. The lowest number of cases was recorded in the children aged 17 and 18 years (Figure 1).

### 3.4. Distribution of Varicella Cases by Vaccination Status

**Figure 2:**
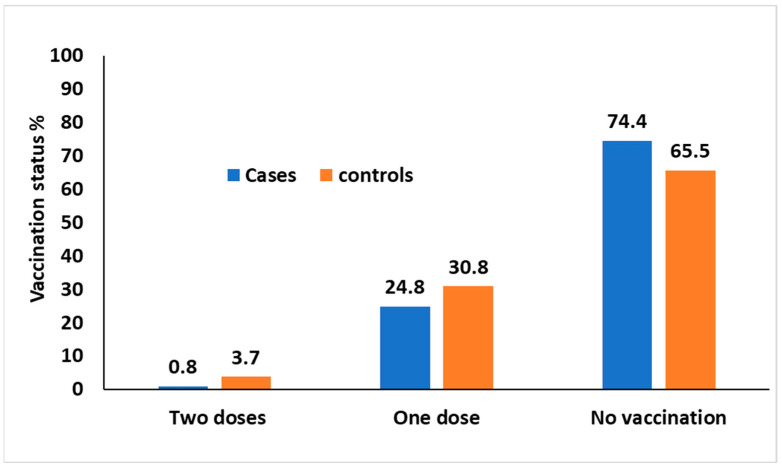
Percentages of cases and controls, distributed by vaccination status.

Among the 862 cases, 7 (0.8%) received two doses of varicella vaccine, 214 (24.8%) received one dose, and 641 (74%) did not receive the vaccine. In contrast, among the 5454 controls, 204 (3.7%) received two doses of vaccine, 1678 (33%) of the controls received one dose, and 3572 (65%) did not receive any vaccination (Figure 2).

### 3.5. Description of the Cases with Two Doses of the Vaccine

Among the 862 cases, there were 7 cases who, despite receiving two doses of the vaccine, contracted a varicella infection. The mean age was 4 years old, with four males and three females. All patients except one had contracted the virus in the year 2019 and all of them were non-Qatari except one (Table 2).

### 3.6. Effectiveness of Varicella Vaccine—Multivariable Logistic Regression

In the multivariable logistic regression analysis (Table 3), after adjusting for age, gender, and nationality, a single-dose vaccination had an estimated 56% reduction in the odds of varicella infection, compared to being unvaccinated (OR 0.44, 95% CI: 0.34–0.55; *p* < 0.000). A full vaccination (two doses), compared to being unvaccinated showed an estimated 87% reduction in the odds of varicella infection (OR 0.13, 95% CI: 0.06–0.29; *p* < 0.000).

## 4. Discussion

In this real-world vaccine effectiveness study, we report several varicella outbreaks in Qatar during the period from 2017 to 2019, with most infections being in the unvaccinated. The vaccine effectiveness of varicella vaccines was 56% and 87% for one- and two-dose regimens, respectively.

Our findings showed that there were several outbreaks and a general increase in reported varicella infection cases in the years 2018 and 2019, compared to 2017. This difference could be explained by the new electronic data management system in Qatar Health’s sector, which was implemented in 2018. A previous study showed that the total number of varicella cases registered in Qatar in 2014 was 574 with an incidence of 259.1 per 100,000 which was higher than in 2012 (244.5) and 2013 (237.4) [16]. These findings indicate that the varicella infection rates are still high, especially in children in the 0–18 years age group but the cause is still unknown.

The study period coincided with an increasing influx of expatriates into Qatar, related to the FIFA World Cup preparations, and this may have contributed to the increase in varicella outbreaks. The varicella vaccines are not mandatory or given as part of routine childhood vaccines, and this could have contributed to the number of unvaccinated children. It may be advisable to consider varicella vaccinations at school entry to reduce future outbreaks. Furthermore, in the case of an influx of expatriates with children is expected, immunization records of children entering any country should be checked, confirmed, and updated by the local ministry of public health. The current program in Qatar ensures that all expatriates arriving in Qatar have to go through a medical commission set up by the government of Qatar where the immunization records are evaluated and updated if any vaccines are missed by any age group. Programs such as these might help in preventing and reducing outbreaks.

In this real-world vaccine effectiveness study, the half dose (single dose) showed a vaccine effectiveness of 56% and the full dose (double dose) varicella vaccination showed an 87% vaccine effectiveness against varicella infection. These results are comparable to the vaccine efficacy ranging from 87% to 95% that has been reported from randomized controlled trials [18,28,29,30,31,32] and the 84% to 98% reported from real-world studies [2,16,31,32].

A similar matched case–control study conducted in Qingdao, China, showed a 44.7% vaccine effectiveness for one-dose and an 81.6% for two-dose vaccinations. It is possible that the low one-dose vaccine effectiveness compared to ours is due to the age group included in this study (6–11) which is reflective of the fact that one dose of the varicella vaccine does not induce an adequate immune response for lifelong immunity. By the age of 5, immunity wanes and hence a second dose is required to boost the immune system [33].

Another similar case–control study in Navarre, Spain, showed a much higher vaccine effectiveness of 87% and 97% for one and two doses, respectively. Compared to our study, there could be several reasons for the discrepancies with our study such as the diagnostic method of laboratory confirmation utilized the other study. In contrast, the data we received for varicella patients were of those who were diagnosed with varicella infection on a clinical basis. Hence, there could have been a possibility that patients with a rash manifestation similar to varicella might have been regarded as cases whereas they might have not had the infection, diluting the effect of our vaccine [33].

The effectiveness in our study may have been lower because of several reasons. During data collection, for the majority of cases and control, we did not receive any details regarding their vaccinations. This point could be a source of weakness in our study, although we believe its effect would be minimal. The unknown vaccination status typically refers to those participants who did not have any dates of vaccination. Varicella vaccination in Qatar was started in 2002 and thus, we can almost certainly say that they were all non-vaccinated. The reason these participants were labeled as unknown in our dataset is that there is a small possibility that the expatriate population who had arrived in Qatar did not have any proof of vaccination and as such, the physicians labeled them as unknown instead of non-vaccinated due to the ambiguity of the information. However, this issue is confined to a very few cases as Qatar has a very strict protocol and mechanism of recording dates of vaccinations, which are stored within the digital health record system and with the parents in the form of a health card. Therefore, this makes it extremely unlikely for vaccinated people in Qatar to have appeared without any vaccination dates in our dataset, further strengthening the point that the unknown criterion represents those who were not vaccinated.

Other studies made use of a national immunization information system, which was already in place and properly utilized by the physicians well before the research started [31]. However, the state of Qatar started to digitally store medical records in 2018, prior to which, all records were physically taken and recorded, which could result in a lack of accuracy in the national dataset in terms of vaccination records.

Among the strengths of the study is that we utilized all the available cases that were in the Cerner dataset and used matched controls. However, the study has some limitations. As described previously, the diagnosis of varicella was through the clinical presentation of rash and not confirmed by serology; this could have led to non-varicella patients presenting with rash similar to varicella also being labeled as varicella-positive, and hence, categorized as false cases for our study, which could dilute and decrease the effectiveness of our study. However, this is common practice in many health systems and expected in a real-world study. Additionally, the study only included children who came to the PHCC with a rash. Thus, the reported data do not capture the cases that did not seek medical care nor those cases presenting with an atypical varicella presentation. Furthermore, in health systems like Qatar, universal access to healthcare is equal. However, it is possible that mild cases may not be reported. Those could include patients who are unvaccinated since they might not be able to seek medical care.

A possible weakness in our study could have been the introduction of recall bias related to the exposure and the vaccination status. A small possibility is that parents might have forgotten the vaccination history of their children. However, due to the stringent mechanism of recording vaccination dates within the health system in Qatar as well as on the vaccination card that is given to the parents, it is extremely unlikely that information regarding vaccination history may have been inaccurate. A small risk might occur for those who had forgotten their vaccination cards; however, we have no reason to believe that the recall bias would be a differential across cases and control.

Our study demonstrates the need for international digitized transferable public health data, such as vaccination records, to ensure correct data keeping regarding vaccination status in all countries all over the world, especially of the expatriate population. Given the global nature and the proliferation of movement, international standardized transferable health data, such as the ones used during COVID-19, can play a major role in alleviating problems such as inaccurate vaccination histories. With the advancement of health record digitization and increased use of internet and smart devices globally, vaccination records are expected to be well kept and easily tracked in the near future, especially the implementation of lessons learned from the COVID-19 pandemic and the worldwide vaccination campaigns to combat the spread of SARS-CoV-2 infections. The pandemic disrupted various immunization programs, mainly pediatric programs in developed and low- and middle-income countries (LMIC), which highlighted the crucial need for proper digital immunization documentation. During the pandemic, COVID-19 immunization records or vaccination status was digitally monitored in many countries, which enhanced the capabilities to maintain digital records [34]. Awareness towards vaccines’ role in preventing infections was highlighted during the COVID-19 pandemic in spite of the misinformation campaign targeting the value of immunization. In the coming years, we expect to witness enhancement in vaccination program logistics and record keeping, which will allow for accurate real-world vaccine efficacy assessment.

## 5. Conclusions

The vaccine effectiveness of varicella vaccines were 56% and 87% for one- and two-dose regimens, respectively. The odds of infection by the varicella virus is diminished by the use of both one-dose and two-dose varicella vaccine regimens. However, a greater odds reduction was conferred by the two-dose regimen compared to the one-dose regimen. Therefore, in this multicultural setting in Qatar, the two-dose varicella vaccination shows reasonable protection against varicella infection. In addition, we suspect that the varicella incidence in Qatar will be significantly reduced since there is a new vaccination program launched by PHCCs. This program aims to raise awareness about the importance of varicella vaccines and their effectiveness.

## Figures and Tables

**Table 1:** Baseline characteristics of the study cohort.

	Controls	Cases	*p* Value
**Total number**	5454	862	
**Age (Median) (IQR) (year)**	8.0 (3–12)	8 (3–12)	0.34
**Age group, n (%)**			0.67
1–5 years	2108 (39)	322 (37)	
6–10 years	1582 (29)	249 (29)	
11–18 years	1764 (32)	291 (34)	
**Gender, n (%)**			0.93
Female	2596 (47.6)	409 (47.4)	
Male	2858 (52.4)	453 (52.6)	
**Nationality, n (%)**			0.012
Qatari	2832 (52)	408 (47)	
Non-Qatari	2622 (48)	454 (53)	
**Visit year, n (%)**			<0.001
2017	1501 (28)	63 (7.3)	
2018	1794 (33)	383 (44)	
2019	2159 (40)	416 (48)	
**Vaccination status, n (%)**			<0.001
No vaccination	3572 (65)	641 (74.4)	
One dose	1678 (33)	214 (24.8)	
Two doses	204 (3.7)	7 (0.8)	

**Table 2:** Description of the 7 cases that were infected with varicella despite having 2 doses of the vaccine.

Case	Gender	Age (Years)	Age Group	Year of Visit	Month of Visit	Health Center	Nationality
1	Male	7	Group 2 (6–10)	2019	11	UMS Umm Slal	Non-Qatari
2	Female	4	Group 1 (1–5)	2019	11	GHR Gharrafat	Non-Qatari
3	Female	4	Group 1 (1–5)	2019	11	ABS Abu Baker	Non-Qatari
4	Male	4	Group 1 (1–5)	2019	12	OBK Al Khatab	Qatari
5	Male	4	Group 1 (1–5)	2018	11	ABN Abu Nakhla	Non-Qatari
6	Female	4	Group 1 (1–5)	2019	3	WAJ AL Wajbah	Non-Qatari
7	Male	5	Group 1 (1–5)	2019	12	GHR Gharrafat	Non-Qatari

**Table 3:** Effectiveness estimates for the overall analysis and for stratified analyses by age groups, gender, nationality, and calendar year.

	Single Dose, OR * (95%CI)	*p*-Value	2 Doses, OR * (95%CI)	*p*-Value
Overall efficacy estimate	0.44 (0.34–0.55)	0.000	0.13 (0.06–0.29)	0.000
Age groups				
1–5 years	0.50 (0.38–0.66)	0.000	0.14 (0.06–0.32)	0.000
6–10 years	0.28 (0.15–0.51)	0.000	0.30 (0.04–2.26)	0.240
11–18 years	9.22 × 10^−7^ (0–.)	−0.03	8.71 × 10^−7^ (0–.)	−0.01
Gender				
Males	0.47 (0.34–0.64)	0.000	0.14 (0.05–0.39)	0.000
Females	0.40 (0.27–0.57)	0.000	0.13 (0.04–0.42)	0.001
Nationality				
Qatari	0.38 (0.26–0.55)	0.000	0.04 (0.01–0.30)	0.002
Non-Qatari	0.48 (0.35–0.66)	0.000	0.21 (0.09–0.51)	0.000
Calendar year				
2017	0.65 (0.20–2.13)	0.475	8.74 × 10^−8^ (0–.)	0.992
2018	0.46 (0.29–0.73)	0.001	0.04 (0.00–0.28)	0.001
2019	0.42 (0.29–0.62)	0.000	0.22 (0.08–0.57)	0.002

* OR: Odds ratio refers to the odds of varicella infection if either 1 dose or 2 doses were received compared to no vaccination.

## Data Availability

Data are provided under MTA with Primary Health Care Centers (PHCC https://www.phcc.gov.qa/, accessed on 29 March 2021) in Qatar and cannot be shared.

## Supplementary Materials

The following supporting information can be downloaded at: https://www.mdpi.com/article/10.3390/vaccines11101567/s1, Table S1: The cases distribution according to the geographical location of each Primary Health Care Center (PHCC) in Qatar; Table S2: Nationalities of the cases according to the geographical regions.

## References

[1] Arvin A.M. (1996). Varicella-zoster virus. Clin. Microbiol. Rev..

[2] Varela F.H., Pinto L.A., Scotta M.C. (2019). Global impact of varicella vaccination programs. Hum. Vaccines Immunother..

[3] Marin M., Seward J.F., Gershon A.A. (2022). 25 Years of Varicella Vaccination in the United States. J. Infect. Dis..

[4] Centers for Disease Control and Prevention (2021). Varicella Vaccination Information for Healthcare Professionals. https://www.cdc.gov/vaccines/vpd/varicella/hcp/index.html#:~:text=CDC%20recommends%202%20doses%20of,4%20through%206%20years%20old.

[5] Al-Turab M., Chehadeh W. (2018). Varicella infection in the Middle East: Prevalence, complications, and vaccination. J. Res. Med. Sci..

[6] Moro P.L., Leung J., Marquez P., Kim Y., Wei S., Su J.R., Marin M. (2022). Safety Surveillance of Varicella Vaccines in the Vaccine Adverse Event Reporting System, United States, 2006–2020. J. Infect. Dis..

[7] Kota V., Grella M.J. (2023). Varicella (Chickenpox) Vaccine. StatPearls.

[8] Stefanizzi P., Stella P., Ancona D., Malcangi K.N., Bianchi F.P., De Nitto S., Ferorelli D., Germinario C.A., Tafuri S. (2019). Adverse Events Following Measles-Mumps-Rubella-Varicella Vaccination and the Case of Seizures: A Post Marketing Active Surveillance in Puglia Italian Region, 2017–2018. Vaccines.

[9] Stefanizzi P., De Nitto S., Patano F., Bianchi F.P., Ferorelli D., Stella P., Ancona D., Bavaro V., Tafuri S. (2020). Post-marketing surveillance of adverse events following measles, mumps, rubella and varicella (MMRV) vaccine: Retrospecive study in apulia region (ITALY), 2009–2017. Hum. Vaccin. Immunother..

[10] Ahn K.H., Park Y.-J., Hong S.-C., Lee E.H., Lee J.-S., Oh M.-J., Kim H.-J. (2016). Congenital varicella syndrome: A systematic review. J. Obstet. Gynaecol..

[11] Al-Dahshan A., Hammoud H., Chehab M., Osman S.R.O. (2019). Vaccination coverage in Qatar: Benchmarking with global figures. Qatar Med. J..

[12] The Peninsula (2019). Qatar Tops Region in Coverage of Quality Vaccines. https://thepeninsulaqatar.com/article/24/04/2019/Qatar-tops-region-in-coverage-of-quality-vaccines.

[13] MOPH (2021). Varicella (Chicken Pox). https://www.moph.gov.qa/english/mediacenter/HealthTips/Pages/HealthTipsDetails.aspx?ItemId=25.

[14] Huang J., Wu Y., Wang M., Jiang J., Zhu Y., Kumar R., Lin S. (2022). The global disease burden of varicella-zoster virus infection from 1990 to 2019. J. Med. Virol..

[15] Hanquet G., Valenciano M., Simondon F., Moren A. (2013). Vaccine effects and impact of vaccination programmes in post-licensure studies. Vaccine.

[16] Wutzler P., Bonanni P., Burgess M., Gershon A., Sáfadi M.A., Casabona G. (2017). Varicella vaccination–the global experience. Expert. Rev. Vaccines.

[17] Spackova M., Wiese-Posselt M., Dehnert M., Matysiak-Klose D., Heininger U., Siedler A. (2010). Comparative varicella vaccine effectiveness during outbreaks in day-care centres. Vaccine.

[18] Shapiro E.D., Vazquez M., Esposito D., Holabird N., Steinberg S.P., Dziura J., LaRussa P.S., Gershon A.A. (2011). Effectiveness of 2 doses of varicella vaccine in children. J. Infect. Dis..

[19] Marin M., Marti M., Kambhampati A., Jeram S.M., Seward J.F. (2016). Global Varicella Vaccine Effectiveness: A Meta-analysis. Pediatrics.

[20] Centers for Disease Control and Prevention (2022). Chickenpox Vaccine Saves Lives Infographic. https://www.cdc.gov/chickenpox/vaccine-infographic.html.

[21] Bansal D., Abdulmajeed J., Al-Shamali M.H.M.A., Albayat S.S.A., Himatt S.M., Cyprian F.S., Chivese T., Mundodan J.M.A., Khogali H.S., Baaboura R. (2022). Duration of COVID-19 mRNA Vaccine Effectiveness against Severe Disease. Vaccines.

[22] Andrews N., Stowe J., Kirsebom F., Toffa S., Rickeard T., Gallagher E., Gower C., Kall M., Groves N., O’connell A.-M. (2022). Covid-19 Vaccine Effectiveness against the Omicron (B.1.1.529) Variant. N. Engl. J. Med..

[23] Castilla J., Cenoz M.G., Abad R., Sánchez-Cambronero L., Lorusso N., Izquierdo C., Llabrés S.C., Roig J., Malvar A., Carril F.G. (2023). Effectiveness of a Meningococcal Group B Vaccine (4CMenB) in Children. N. Engl. J. Med..

[24] Planning and Statistics Authority Home Page (2022). Qatar Monthly Statistics. https://www.psa.gov.qa/en/Pages/default.aspx.

[25] Tayla Holman K.L. (2018). Cerner Corp.. https://www.techtarget.com/searchhealthit/definition/Cerner-Corp.

[26] Cerner O. (2022). Hamad Medical Corporation and Primary Health Care Corporation Adopt the Cerner Managed Technology to Deliver Better Outcomes in Qatar. https://www.cerner.com/ae/en/blog/hmc-and-phcc-adopt-the-cerner-managed-technology-to-deliver-better-outcomes-in-qatar.

[27] Ayoade F., Kumar S. (2022). Varicella Zoster. StatPearls.

[28] Casabona G., Habib A., Povey M., Bergsaker M.A.R., Flodmark C., Espnes K.A., Tøndel C., Silfverdal S. (2022). Randomised controlled trial showed long-term efficacy, immunogenicity and safety of varicella vaccines in Norwegian and Swedish children. Acta Paediatr..

[29] Hao B., Chen Z., Zeng G., Huang L., Luan C., Xie Z., Chen J., Bao M., Tian X., Xu B. (2019). Efficacy, safety and immunogenicity of live attenuated varicella vaccine in healthy children in China: Double-blind, randomized, placebo-controlled clinical trial. Clin. Microbiol. Infect..

[30] Cenoz M.G., Martínez-Artola V., Guevara M., Ezpeleta C., Barricarte A., Castilla J. (2013). Effectiveness of one and two doses of varicella vaccine in preventing laboratory-confirmed cases in children in Navarre, Spain. Hum. Vaccin. Immunother..

[31] Shu M., Zhang D., Ma R., Yang T., Pan X. (2022). Long-term vaccine efficacy of a 2-dose varicella vaccine in China from 2011 to 2021: A retrospective observational study. Front. Public Health.

[32] Vázquez M., LaRussa P.S., Gershon A.A., Steinberg S.P., Freudigman K., Shapiro E.D. (2001). The Effectiveness of the Varicella Vaccine in Clinical Practice. N. Engl. J. Med..

[33] Hu P., Yang F., Li X., Wang Y., Xiao T., Li H., Wang W., Guan J., Li S. (2021). Effectiveness of one-dose versus two-dose varicella vaccine in children in Qingdao, China: A matched case-control study. Hum. Vaccines Immunother..

[34] Ho L.L., Gurung S., Mirza I., Nicolas H.D., Steulet C., Burman A.L., Danovaro-Holliday M.C., Sodha S.V., Kretsinger K. (2022). Impact of the SARS-CoV-2 pandemic on vaccine-preventable disease campaigns. Int. J. Infect. Dis..

