# Supplementary material for: Real-World Effectiveness of the Varicella Vaccine among Children and Adolescents in Qatar: A Case–Control Study
Source: Vaccines (Basel). 2023 Oct 5;11(10):1567. doi: 10.3390/vaccines11101567 (PMC10611158; doi:10.3390/vaccines11101567)
Supplement: Supplementary file 1 [file vaccines-11-01567-s001.zip › vaccines-2539616-supplementary.pdf]

Real-World Effectiveness of the Varicella Vaccine among Children and Adolescents in Qatar: A Case–Control Study

Zahra Bibi 1,†, Ahmed Daniyal Nawaz 1,†, Maha Al Kurbi 1, Shahad Fakhroo 1, Khaled Ferih 1, Noor Aljaber 1, Merin Alex 2, Khalid H. Elawad 2, Tawanda Chivese 1 and Susu M. Zughaier 1,*

Supplementary material file

Supplementary Table S1: Cases distribution in PHCC

Health center	2017	2018	2019	Total Freq(%)	1-18 Years old PHCC visited patients in 2017-2019	Chicken Pox cases per PHCC visited patients (%)
ABN Abu Nakhla	2	14	19	35 (4.1)	16359	0.21
ABS Abu Baker	0	34	41	75 (8.7)	25775	0.29
ARP Airport	0	10	3	13 (1.5)	19202	0.07
DYN Al Daayen	0	4	3	7 (0.8)	12440	0.06
GHR Gharrafat	10	43	26	79 (9.2)	29355	0.27
GHW Leghwairiya	0	1	0	1 (0.1)	554	0.18
JUM Jumailiya	0	0	1	1 (0.1)	683	0.15
KHR Al Khor	6	18	2	26 (3.0)	12015	0.22
LBB Leabaib	0	27	39	66 (7.7)	21960	0.30
MES Mesaimeer	0	22	36	58 (6.7)	41747	0.14
MKH M-Khalifa	0	24	12	36 (4.2)	18823	0.19
MUA Muaither	0	19	39	58 (6.7)	18033	0.32
OBK Al Khatab	17	18	23	58 (6.7)	12798	0.45
QUN Qatar Univ	0	0	6	6 (0.7)	5342	0.11
RAK Rawdat	0	19	31	50 (5.8)	30833	0.16
RUW Al Ruwais	0	1	2	3 (0.3)	2408	0.12
RYN Al Rayyan	0	13	13	26 (3.0)	23474	0.11
SHN Sheehaniya	24	23	20	67 (7.8)	8510	0.79
THM Al Thumama	0	13	19	32 (3.7)	19156	0.17
UMG Um Ghwalina	0	7	10	17 (2.0)	13197	0.13
UMS Umm Slal	0	23	16	39 (4.5)	17973	0.22

WAB Al Waab	0	3	10	13 (1.5)	9433	0.14
WAJ AL Wajbah	0	9	18	27 (3.1)	11878	0.23
WAK Al Wakra	0	21	12	33 (3.8)	34765	0.09
WBC West Bay	4	17	15	36 (4.2)	14727	0.24
Total	63	383	416	862	423156	0.20

Table S2

Supplementary Table S2: Nationalities of the cases according to the geographical regions.

**Region/ nationality**	**Freq. (n)**	**Percent (%)**
**Asia**		
Bangladeshi	10	1.16
Filipino	13	1.51
Indian	78	9.05
Pakistani	58	6.73
Sri Lankan	2	0.23
**Middle East & Africa**		
Bahraini	2	0.23
Egyptian	86	9.98
Iranian	11	1.28
Iraqi	2	0.23
Palestinian	23	2.67
Jordanian	29	3.36
Lebanese	5	0.58
Omani	6	0.70
Qatari	408	47.33
Saudi	14	1.62
Syrian	27	3.13
Yemeni	35	4.06
**Africa**		

Algerian	2	0.23
Eritrean	1	0.12
Ethiopian	2	0.23
Moroccan	1	0.12
Nigerian	3	0.35
Somali	2	0.23
Sudanese	18	2.09
Tunisian	7	0.81
**Europe**		
British	6	0.70
**Americas**		
American	9	1.04
Canadian	2	0.23
**Total**	862	100

Supplementary Figure S1

Qatar Immunization services: link: https://www.moph.gov.qa/english/OurServices/eservices/Pages/Baby-Immunization%E2%80%8B%E2%80%8B.aspx

IMMUNIZATION SCHEDULE
IN THE STATE OF QATAR 2019

AGE / VACCINE	BIRTH	2 MONTHS	4 MONTHS	6 MONTHS	12 MONTHS	18 MONTHS	2 YEARS	4-6 YEARS	13-18 YEARS
BCG	BCG								
HEPATITIS B	Hepatitis B								
PENTA				Penta					
HEXA		Hexa 1	Hexa 2						
TETRA						Tetra *			
DTaP								DTaP B**	
Tdap									Tdap
ROTAVIRUS		Rota 1	Rota 2						
OPV				OPV 1		OPV 2		OPV 3	
PCV 13		PCV 1	PCV 2	PCV 3					
MMR					MMR 1	MMR 2			
VARICELLA					Varicella 1			Varicella 2	
HEPATITIS A					Hepatitis A (2 doses with 6 months interval)				
MENINGOCOCCAL ACYW135							Meningococcal		
INFLUENZA				Influenza					

- (Black) Routinely recommended vaccines.
- (Orange) Influenza vaccine recommended annually from 6 months and above during flu season especially for high risk persons.
- (Blue) Meningococcal vaccine is recommended for children at 2 years of age, high risk persons and travelers to endemic areas
- (Red) Two doses of Hepatitis A vaccine is recommended for children and adolescents with frequent travel to high risk countries.

1. **MMR**: Measles, Mumps, Rubella
2. **HEXA**: Hepatitis B, DTaP, Hib, IPV
3. **PENTA**: Hepatitis B, DTP, Hib
4. **TETRA**: DTaP, Hib

* DTaP alone or Pentaxim or any vaccine containing DTaP may be used at 18 months if Tetra is not available. DTaP/DTP/DT vaccine should not be used above 6 years of age. Use Tdap/Td instead.

** Tdap (Boostrix brand) can be used if DTaP is not available.

For more information, Please call
Hotline: 66740948 - 66740951

www.moph.gov.qa /MOPHQatar /MOPHQatar
